# Zarit Burden Interview Psychometric Indicators Applied in Older People Caregivers of Other Elderly**^1^**

**DOI:** 10.1590/1518-8345.1379.2835

**Published:** 2016-11-28

**Authors:** Mariana Bianchi, Leticia Decimo Flesch, Erika Valeska da Costa Alves, Samila Sathler Taveres Batistoni, Anita Liberalesso Neri

**Affiliations:** 2 Doctoral student, Faculdade de Ciências Médicas, Universidade Estadual de Campinas, Campinas, SP, Brazil. Scholarship holder at Coordenação de Aperfeiçoamento de Pessoal de Nível Superior (CAPES), Brazil.; 3 Doctoral student, Faculdade de Ciências Médicas, Universidade Estadual de Campinas, Campinas, SP, Brazil. Scholarship holder at Coordenação de Aperfeiçoamento de Pessoal de Nível Superior (CAPES), Brazil. Assistant Professor, Faculdade Metropolitana da Grande Fortaleza, Ceará, Brazil.; 4 PhD, Assistant Professor, Escola de Artes, Ciencias e Humanidades, Universidade de São Paulo, São Paulo, SP, Brazil.; 5 PhD, Full Professor, Faculdade de Ciências Médicas, Universidade Estadual de Campinas, Campinas, SP, Brazil.

**Keywords:** Caregivers, Aged, Stress Psychological, Depression, Geriatrics

## Abstract

**Objective:**

to derive psychometric indicators of construct validity and internal consistence of the Zarit Burden Interview scale for caregivers, describing associations of the scale with metrics related to care demands, coping strategies and depression in aged caregivers.

**Method:**

crosscutting descriptive and correlational study. The convenience sample was composed by a hundred and twenty one senior caregivers (Avg=70.5 ± 7.2 years, 73% women). They answered a questionnaire to check the physical and cognitive demands of care, the Zarit Burden Interview (ZBI), the California Inventory of Coping Strategies and the Geriatric Depression Scale (GDS-15).

**Results:**

ZBI showed good internal consistency and also for the three factors emerging from factor analysis, explaining 44% of variability. ZBI is positively related with objective care demands (p < 0.001), depression (p = 0.006) and use of dysfunctional coping strategies (p = 0.0007).

**Conclusion:**

ZBI is of interest to be applied to aged caregivers and the association of higher degrees of burden, dysfunctional coping and depression show a vulnerability scenario that may affect to older people taking care of other elderly.

## Introduction

The gerontology literature exposes the burden perception reported by caregivers of older people, as an important variable in understanding the health outcomes of the caregiver and the quality of care provided^1^. The perceived overload is a psychological indicator designating the attitudes and emotional responses of the caregiver facing the demands of caring. It is considered a multidimensional and multifaceted concept^2^, involving negative cognitive evaluations related to the context and the provision of care and changes in the wellness state and the self^3^.

Being essentially the result of subjective evaluation, the perceived burden is affected by a number of other conditions and assessments, such as the number of care demands, changes in routines and roles, and expectations of outcomes. In particular, the assessment of available resources to exercise care and coping strategies used by caregivers can influence the sense of capacity of the caregiver to meet the demands of activities, minimizing or enlarging the perception of burden^4^.

Caring for older people and assuming the role of caregivers expose people of all ages to the chance of feeling overburdened. However, when an old person assumes this role, it configures a peculiar scenario where demands and variables related to the process of aging of the caregivers interact with the stress originated from the care situation. The elderly caregiver and their peculiarities such as: overload perceptions, coping resources and the description of their well-being levels, are still scarcely explored in the research literature. For this reason there is insufficient evidence to guide the care to the aged population and consequently, there is no self-report measures specially developed especially for this age group of caregivers.

To that end, this study sought to achieve two main objectives. The first one refers to identify indicators of the construct validity and internal consistency of the instruments most used in Brazil to measure the overload in caregivers of older people, namely the Zarit Burden Interview (ZBI)^5^ ^-^ ^6^. It is also important to elucidate through psychometric examination the possible peculiarities in the burden construct when reported by older people caregivers who care for other elderly with different demands, and not only in the context of Alzheimer's disease, its most common use. A second objective of the study is to describe a sample of older people caregivers and identify associations between socio-demographic characteristics, care demands, overload, coping strategies and depression, with the aim to expand the evidence and contributions to this subject in Brazil. 

## Method

This is a descriptive, cross-sectional and correlational study from partial data coming from the study called "Psychological well-being of older people caring for other elderly in the family context", performed by a group of researchers from the Graduate Program of Gerontology, Unicamp. The research was applied to a sample of 121 caregivers recruited by referral from practitioners linked to public and private services for the elderly, and conducted in four counties in the state of Sao Paulo. The research included caregivers aged 60 and over, who were informally in charge of care to an elderly family member in the home context and with some degree of dependency, for six months or more and, as well as agreeing to participate. Caregivers were excluded when presented suspected cognitive decline according to standards established by the cognitive screening tool CASI-S (Cognitive Abilities Screening Instrument - Short Form)^7^ for use in Brazil. Data collection was conducted from October 2014 to July 2015, after approval by the Ethics Committee of the State University of Campinas (CAAE: 35868514.8.0000.5404). After signing the Informed Consent Form by the caregiver, the interviews were conducted by trained researchers in the health services or in the caregivers' homes according to the preference of the respondent, with an average duration of 60 minutes. At the end of the interview, it was offered to the caregivers an informative manual on communication strategies with older people, developed by the researchers, as a token of gratitude for their participation. 

For the present study, we extracted from the largest protocol, the following variables and instruments:

a) Socio-demographic data and related to the caregivers' role: to characterize the sample in terms of gender, age, education, income, co-habitation, family ties with the elderly, time exercising care and if they are the primary caregivers.
b) Care demands: to survey the level of aid in activities of daily living and cognitive nature of demands. b.1) Aid intensity: identified from an adaptation of Daily Life Activities Inventory^8^, activities such as bathing, dressing, using the toilet, transfer, toilet training and feeding and daily life Instrumental Activities^9^, such as telephone use, transportation, shopping, food preparation, housekeeping, medication use and management of money. After completing each item above referred, was added to the question "Are you the main source of help in this task?" assigning a point for each affirmative answer. Thus, the aid intensity could vary from 0 to 13 points depending on how much help is spent by the caregiver. b.2) Cognitive demands: identified from the application of the Clinical Dementia Rating (CDR)^10^. Originally used for screening and staging of dementia, it was used in this study to assess the caregiver about the cognitive functionality of dependent elderly in the areas of memory, orientation, judgment and problem solving, community relations, home and hobbies and personal care, generating the following interpretation for their scores: 0 = normal, 0.5 = questionable, 1 = mild, 2 = moderate, 3 = severe.
c) Perceived burden: identified by Zarit Burden Interview (ZBI), a scale made up by 22 items with five points each (0 = not at all to 4 = always), ranging from 0 to 88, a score that reflects the burden level of caregivers, where the higher the score, the greater is the perceived overload. The ZBI was validated in Brazil with an older people caregivers sample with psychiatric disorders, by Scazufca and colleagues^6^ and this study aims to research psychometric indicators when applied to older people caring for other seniors with various kinds of dependency.
d) Inventory of Coping Strategies^11^: designed specifically for seniors to investigate the ways in which they react to situations that carry special demands on the adaptive resources, requiring the adoption of strategies to cope with internal and external requirements that characterize such situations and thus adjust to them. It has been validated in Brazil^12^ and consists of 19 items of four points (never = 0, occasionally = 1, often = 2, always = 3). The score is made from the weighted averages in the factors or domains that comprise it. For the present study will be calculated the average in the areas identified by the methodological study^13^ as follows: 1. dysfunctional strategies concerning avoidance or behavioral excesses strategies, not beneficial to coping with the caregiving situation (e.g. "to use medicines to control anxiety, "drinking and "overeating", "demonstrate hostility"); 2. selective secondary control strategies, involving strategies that are not intended to direct action on the stressor (in the case, care), but triggers social or personal nature resources that motivate coping; 3. compensatory secondary control strategies, involving strategies of indirect perception of control on the situation, triggering resources from external or spiritual sources (example: "pray", "trust in God" or "try to forget about the problem").
e) Geriatric Depression Scale: The Geriatric Depression Scale (GDS-15) is a dichotomous scale for the presence or absence identification of symptoms related to changes in mood and specific feelings such as helplessness, worthlessness, disinterest, boredom and happiness. In Brazil, the cut point larger or equal to six points is adopted following a study scale validation performed by Almeida and Almeida^14^.

For the analysis of construct validity and internal consistency indicators of the ZBI we used exploratory factor analysis in order to analyze the composition of factors with the method of estimation of main components. To fix the number of factors was used the screeplot test. It was then applied the rotation of factors through orthogonal Varimax method. Items with a load higher than 0.30 were considered in one of the rotated factors for combination of factors. To analyze the internal consistency of the resulting factors and the total scale was used Cronbach's alpha.

For further analysis of the study were used the chi-square or Fisher's exact test (for expected values lower than 5), the Mann-Whitney test to compare the numerical variables between two groups, and the Kruskal-Wallis test to compare the numerical variables between three or more groups. The total score and factors of ZBI, the Coping Inventory and GDS-15 were submitted to Spearman correlation to analyze possible correlations between these variables and ZBI. Analyses were performed using the computer statistical program SAS for Windows (Statistical Analysis System), version 9.2.

## Results

The sample of 121 caregivers consisted mostly of women (73%), average age of 70.5 years (SD = 7.20), married (83%), gross household income on average, 4.3 minimum wages (SD = 3.79) and 5.8 years of education (SD = 4.32). Regarding the relationship to the elderly, 62% were spouses, followed by caregivers with other ties such as parents, in-laws, siblings, uncles and children. Most caregivers (84%) live with the subject of care. The average time performing the role of caregiver was 5.34 years (SD = 6.27), ranging from six months to 40 years of care. There were no differences in gender of participants related to other socio-demographic variables and related care.

Concerning the scope of the first objective of the study, the ZBI items revealed, from the measurement of Kaiser MSA (>0.60) consistency, allowing to be used for the exploratory factor analysis. Through the factors selection criteria with eigenvalues greater than 1, we obtained 8 factors, which explained 71.5% of the variability of the data. Through the screeplot test, it was decided to fix the extraction of three factors, which explained 44.0% of the total variability, since from this factor on the curve stabilized without major increases in the accumulated percentage of explained variance. Table 1 shows the charge and composition of three factors resulting after the orthogonal Varimax rotation and the commonality of the scale items. The items 3, 10, 9 and 7 had loads >0:30 in more than one factor, and were placed in the factor with the largest load. Item 20 presented the greatest commonality, i.e. 76.5% of its variability was explained by the factors, and item 14 had the lowest commonality (13.1%). The scale was applied to the elderly showing high internal consistency for all factors and total. It is noteworthy that for the analysis of psychometric indicators of the scale, we used data from 110 caregivers who responded to all the items of the scale, which compared to the total sample (n = 121) revealed no statistically significant differences in other measures.

**Table 1:**
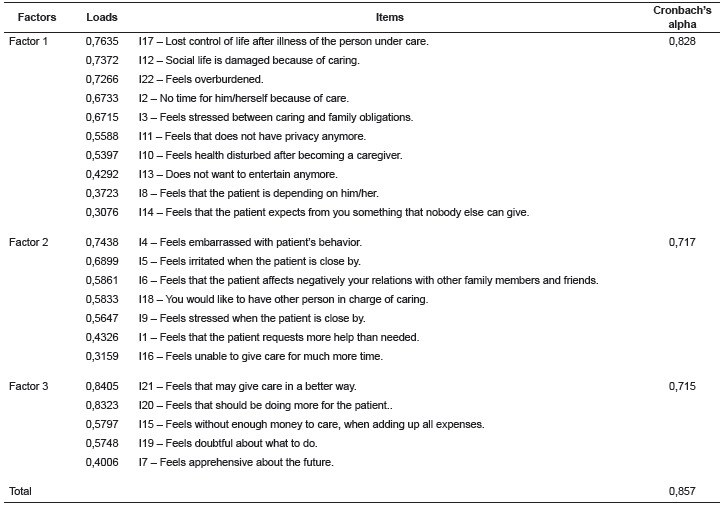
Results of the Exploratory Factor Analysis after orthogonal rotation of the 22 items of the 22 items of the Zarit Burden Interview. Campinas, SP, Brazil, 2015

Table 2 describes the sample according to the burden metrics, coping strategies and depression. To obtain the intensity of the aid offered by caregivers, we divided the distribution of responses in tertiles. The offer of help was considered of high intensity when the score was 8-13 points. It was observed that most caregivers take care of the elderly with mild to questionable degree of cognitive impairment (44.8%).

**Table 2:**
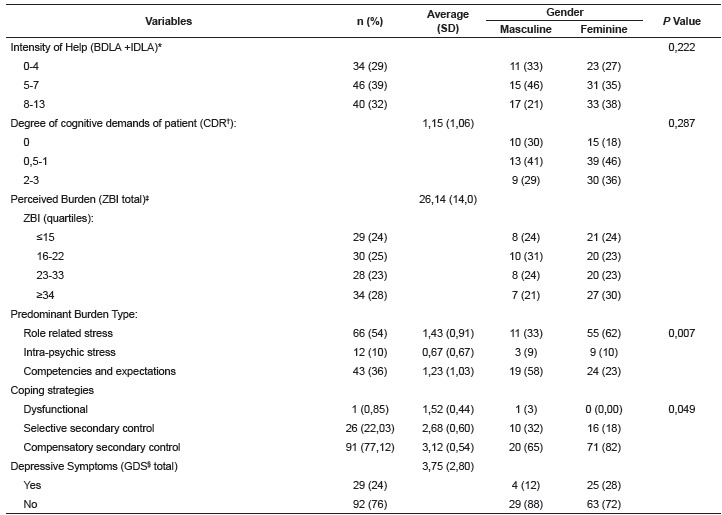
Distribution of frequencies, averages and standard deviation of the variables under study in the whole sample and by gender. Campinas, SP, Brazil, 2015

* BDLA: basic daily living activities, IBDLA: instrumental daily living activities ; † CDR: Clinical Dementia Rating; ‡ ZBI: Zarit Burden Interview; § GDS: Geriatric Depression Scale

Perceived burden as identified by ZBI averaged 26.1 points, ranging from 3 to 80 points. The distribution of the sample into quartiles identified several score ranges. Scores from 23 to 33 points and scores higher than 34 points were categorized as moderate overload and high overload. From the factor composition of the ZBI, the frequency of older people with major average score of the identified factors may be deducted. Factors 1 and 3 showed the highest frequencies.

As referred to the coping strategies, only one individual obtained a high average of strategies for the factor "dysfunctional strategies." In turn, 26 individuals had prevalence in the use of selective secondary control coping strategies, with an average of 2.68 (SD = 0.60), ranging from 1.41 to 4 points. In the factor relating to compensatory secondary control strategies, 91 individuals presented its use, with a mean of 3.12 (SD = 0.54) ranging from 1 to 4 points. About 24% of the sample had scores in the GDS-15 scale suggesting the presence of depressive symptoms. The average score was 3.75 (SD = 2.8), ranging from 0 to 11 points.

Table 3 shows the results of Spearman's correlation analysis.

**Table 3:**
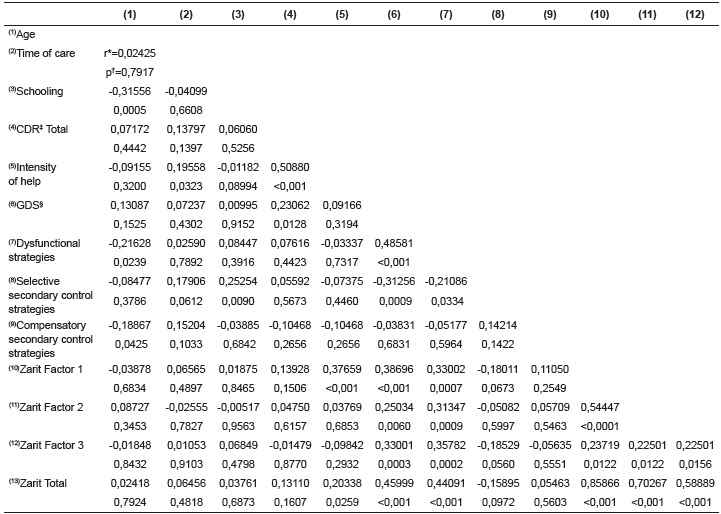
Results of Spearman correlation between the studied variables. Campinas, SP, Brazil, 2015

* Spearman's correlation coefficient; † p value; ‡ Clinical Dementia Rating; § Geriatric Depression Scale

## Discussion 

The socio-demographic description of the research sample replicates some classic data on the role of caregivers by women and spouses^15^. Other aspects are also similar to those found in studies with younger caregivers, however, it is important to pay attention to the challenges posed by the fact that caregivers of the sample are themselves older people. Among these factors there is the socioeconomic reality in which they assume this role and the time extension performing this role that they may experiment. An older people dyad possibly presents higher costs in relation to the cost of health services, medicines and transport that can generate financial difficulties and special stresses, and being in majority spouses, the care generally has its end with the caregivers exhaustion or diseases in their health and functionality or the death of a party

The sample composed according to convenience criteria, has the peculiarity of being caretakers of elderly people with many physical demands and possibly less cognitive demands, as assessed by the degree of impairments, suggested through CDR as being questionable or mild declines. These features are different from the data generated by the large volume of studies developed with younger caregivers and caregivers of patients with Alzheimer's disease^16^. Among younger caregivers and also those who care for older adults with Alzheimer, scores in Zarit Burden Scale (ZBI) also tend to be higher than those found in this study that reached an average of 26.1 points within a possible range 0-88 points^17^. The prevalence rate of depression suggested by the GDS-15 also showed no significant differences related to the rate found in the aged population in general^18^ ^-^ ^19^.

Among younger caregivers, prevalence measures of this condition tend to be higher, since they are exposed to a stressful and chronic condition. However, for the present sample, aspects related to the aging process, such as an increase in psychological resilience in old age, even in the presence of different physical conditions, can be a factor related to the protection of the sample against adverse outcomes in terms of perceived burden and depression. Psychological research with aged population points out the influence of adaptive coping resources such as selective and compensatory strategies of control regarding adverse events. In the sample of this study it was virtually absent the predominant use of dysfunctional and largely it was revealed the use of compensatory strategies to reframe stressful situations through spiritual and existential resources. On the other hand, taking care of the spouse in old age also tend to be experienced, especially among older cohorts, as a normative life event, making it less stressful.

 Taking care of another elderly in old age may represent a different reality than when experienced by younger people, requiring a more specific examination. To test this premise, two analytical objectives have guided the present study. The first sought to describe the psychometric indicators of ZBI when applied to older people caring for other seniors. More than responding to a methodological objective, the good psychometric indicators confirming the use of the scale among caregivers, such scale analysis pointed to the possibility of examining the burden construct in its multi-dimensionality and the possible peculiarities of this construct in aged caregivers. Three possible explanatory domain of this construct were identified from the interpretation of the factors generated and in the light of classical literature on caregiver stress, especially as proposed by Pearlin and colleagues^20^. Thus, the first explanatory domain of the burden construct in the older people was called "Tensions related to the role," since it gathered items on impact on the daily lives of caregivers, such as lack of time, privacy, impairments in social life, health disturbs, feeling of loss of control of life. The second burden construct domain was called "Intra-psychic tensions", as it gathered items related to specific emotional manifestations, as feelings of shame, anger, indecision about care. The third domain referred to the presence or absence of "competencies and expectations" linked to care, i.e. the perception that they should be doing more or taking better care of the elderly patient.

In international studies including samples from different ages of older caregivers, a similar sort of explanatory fields emerged. In a Spanish study, three explanatory factors appeared: impact on care, interpersonal relationships and expectation of self-efficacy^21^. The application of the ZBI in a sample of Portuguese caregivers, however, generated a structure with four factors, that may be (interpretatively) recognized in two core dimensions of objective burden (impact of care and interpersonal relationships) and subjective burden (expectations with care and the perception of self-efficacy)^22^. In this study, unlike the aforementioned studies, the second explanatory domain deals specifically with the emotional impacts, which will be a possible distinctive feature of a sample of older people caring for other seniors. 

Examination of average of frequencies of the elderly in the scale dimensions showed that the dimensions "tensions related to the role" and "competencies and expectations" were significantly larger than the dimension "intra-psychic tensions". It is possible that the fact that looking after another at old age is a more normative occurrence, resulting that most of the sample do not experience psychic stress, burden or depression, as revealed by the low frequency of such conditions^23^.

The second objective of this study was to identify associations between care context variables, burden, coping strategies and depression. Some interesting correlations were found that might help in illuminating the phenomenon of caring in old age. Increasing age was positively associated to the time exercising the role of caregiver. Another socio-demographic variable highlighted in the correlation analysis was education. There was a positive association between years of schooling and selective secondary control strategies. Such strategies refer to resorting to alternative sources of support or help, such as those achieved by economic or social ways. This association suggests that the level of education is a proxy for other resources to face the challenges of care, as was already reported in other studies in Gerontology on stress and coping in old age^24^.

We found positive association between perceived burden and aid intensity. This association may be related to the view that the burden among older people caregivers may refer more to wear and tear in physical demands, as they can be strenuous to the aged body. As expected, there was positive association between negative indicators of care such as: between depression and use of dysfunctional strategies and between depression and total burden and in all domains. The increase in the perception of care demands and the use of dysfunctional strategies has a psychological nature associated with negative outcomes in mental health of caregivers. This is pointed out by meta-analysis on the subject of caregivers, and is not different among older people caregivers^25^. On the other hand, there was a negative correlation between the use of secondary control strategies (either selective or compensatory) and depression, suggesting that they may act as protective or cushioning of the stress of caring.

It should be noted the methodological limitations of the study that may limit the generalization of findings. This is a cross-sectional study that does not allow consistent causal inferences that longitudinal prospective studies may address in the future. It also uses data derived from a convenience sample, recognizing, however, the difficulties in performing studies with random samples on this subject, both in terms of costs and time spent. Noteworthy is also the need for continuity of psychometric studies of ZBI among older people caregivers, especially regarding its construct, using for example confirmatory factor analysis strategies. It is recognized that the study did not control the presence of chronic diseases, very common in the elderly, limiting itself to the examination of burden relations with indicators of assistive demands and psychological health. 

## Conclusion 

The two analytical objectives of the study generated evidence that suggest peculiarities in the study, measurements and interpretation of data collected from older people caregivers. Factor analysis of the ZBI and examination of the internal consistency reached validity indicators allowing the use of the scale with the elderly, but the examination of the scores among their domains suggests that these are probably less affected psychically by the demands and general requirements of care. Thus remarks the opportunity that the scale presents to allow a more refined examination of the caregiver burden beyond the one-dimensional use of the same as reflected by a total score. In general, the study also points to the fact that the elderly take care of other seniors even in the presence of psychological discomforts, such as depression, or in the presence of strenuous physical demands.

The specific examination of elderly caregivers becomes increasingly urgent in the face of socio-structural changes such as aging populations, lower supply of caregivers due to the greater inclusion of women in the labor market and fewer children per couple and also caused by changes in profile of morbidity due to chronic and degenerative diseases that require long-term care in time. On the other hand we need to consider the overlap between the demands of the aging caregiver and the demands and stress generated by the care that can expose the elderly caregiver to a double condition of vulnerability to adverse outcomes in physical and mental health. The gerontology research, health care and public policies should be prepared for the special features in this growing reality.
